# Supplementary material for: Wetting and complex remodeling of membranes by biomolecular condensates
Source: Nat Commun. 2023 May 22;14:2809. doi: 10.1038/s41467-023-37955-2 (PMC10203268; doi:10.1038/s41467-023-37955-2)
Supplement: Supplementary file 3 — Description of Additional Supplementary Files [file 41467_2023_37955_MOESM3_ESM.pdf]

Description of Additional Supplementary Files

Title: Supplementary Movie 1
Description: Wetting dynamics for DOPC GUVs (magenta) in contact with glycinin condensates (cyan) at 100 mM NaCl (see Fig. 4a,b). Timestamps are given in minutes (hh:mm) where zero time corresponds to the initial contact between the droplet and the condensate after mixing them. The field of view is 32.6 μm × 25.9 μm.

Title: Supplementary Movie 2
Description: Z-scan of a GUV-droplet system (DOPC GUV, glycinin at 100 mM) presenting ruffling/fingering of the condensate-membrane interface (see Fig. 6). Field of view is 20.0 μm × 20.0 μm.

Title: Supplementary Movie 3
Description: Ruffling/fingering dynamics (DOPC GUV, glycinin at 100 mM); see Fig. 7a,b. Timestamps on the images are given in hours:minutes (hh:mm). The field of view is 26.9 μm × 26.9 μm.

Title: Supplementary Movie 4
Description: Ruffling/fingering structures do not fluctuate (DOPC GUV, glycinin at 100 mM). Time is given in hours:minutes:seconds (hh:mm:ss). The field of view is 18.9 μm × 18.9 μm.

Title: Supplementary Movie 5
Description: Ruffling/fingering suppression by increasing membrane tension. Micropipette aspiration of a GUV-droplet system (DOPC GUV, glycinin at 100 mM) presenting interfacial ruffling (see Fig. 8). Field of view is 28.9 μm × 28.9 μm.
